# Circularly Polarized Light-Enabled Chiral Nanomaterials: From Fabrication to Application

**DOI:** 10.1007/s40820-022-01005-1

**Published:** 2023-01-18

**Authors:** Changlong Hao, Gaoyang Wang, Chen Chen, Jun Xu, Chuanlai Xu, Hua Kuang, Liguang Xu

**Affiliations:** 1 International Joint Research Laboratory for Biointerface and Biodetection, State Key Lab of Food Science and Technology, School of Food Science and Technology, Jiangnan University, Wuxi, Jiangsu, 214122 People’s Republic of China; 2 Department of Neurology, China National Clinical Research Center for Neurological Diseases, Beijing Tiantan Hospital, Capital Medical University, No.119 South 4Th Ring West Road, Beijing, 100070 People’s Republic of China

**Keywords:** Circularly polarized light, Chiral, Nanomaterials, Fabrication, Application

## Abstract

For decades, chiral nanomaterials have been extensively studied because of their extraordinary properties. Chiral nanostructures have attracted a lot of interest because of their potential applications including biosensing, asymmetric catalysis, optical devices, and negative index materials. Circularly polarized light (CPL) is the most attractive source for chirality owing to its high availability, and now it has been used as a chiral source for the preparation of chiral matter. In this review, the recent progress in the field of CPL-enabled chiral nanomaterials is summarized. Firstly, the recent advancements in the fabrication of chiral materials using circularly polarized light are described, focusing on the unique strategies. Secondly, an overview of the potential applications of chiral nanomaterials driven by CPL is provided, with a particular emphasis on biosensing, catalysis, and phototherapy. Finally, a perspective on the challenges in the field of CPL-enabled chiral nanomaterials is given.

## Highlights

- This review summarized the fabrication strategy using circularly polarized light as a chiral source to construct chiral materials.
- The potential applications of chiral nanomaterials driven by circularly polarized light in different fields are summarized, explained by representative examples.
- The potential challenges of circularly polarized light-enabled chiral materials are outlined and future research directions are outlooked.

## Graphical abstract

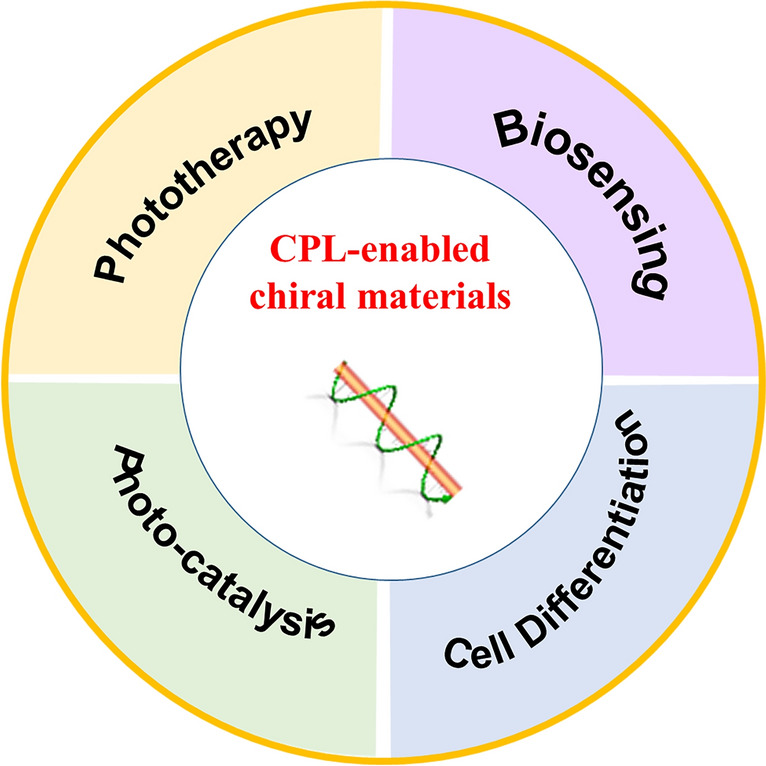

## Introduction

Circularly polarized light (CPL) could be a possible reason for the single handedness of biomolecules. Linearly polarized (LP) light is composed of left- and right-circularly polarized (LCP and RCP) light with equal intensity [1, 2]. The electromagnetic fields of LCP and RCP light are mirror images of each other. The optical properties of chiral materials are usually characterized by circular dichroism and optical rotation. Circular dichroism is the differential absorption of LCP and RCP light. When LCP and RCP lights pass through a medium of chiral material, the interaction of the chiral material with LCP light is not equal to that with RCP light, thus leading to a difference in light velocity and refractive index, and this phenomenon generates optical rotation. As a chiral source, circularly polarized light has attracted considerable attention and has been applied to many fields, such as synthesis of chiral molecules, asymmetric catalysis, dynamic control and amplification of molecular chirality, trigger photochemical reactions, and so on [3–8]. For instance, in order to complete chiral symmetry breaking of the racemates of the amino acid derivative, the racemates were firstly irradiated with CPL (310 nm, 0.3 mW for 70 h) to induce the reaction and a small enantiomeric excess (e.e.) value was obtained. Then ultrasonic grinding was applied to accelerate the process of deracemization due to some sort of a ripening mechanism [9].

Nanomaterials with chiroptical activity are known to strongly rotate the polarization of linearly polarized light. Due to the fast development of chiral nanoscience and nanotechnology, many methods have been developed to prepare the chiral nanomaterials during the past decades. A lot of chiral materials, such as chiral gold nanostructures [10, 11], chiral liquid crystal nanostructures [12–15], chiral cobalt oxide nanoparticles [16], chiral CdTe nanoparticles [17, 18], chiral grapheme quantum dots [19], chiral CdSe nanoparticles [20, 21], chiral HgS nanoparticles [22], chiral ceramic nanoparticles [23], chiral Te nanowires [24], chiral cobalt hydroxide nanoparticles [25], chiral copper sulfide nanoparticles [26], chiral nickel sulfide nanoparticles [27], chiral Au film [28], and chiral Fe_3_O_4_ film [29], and chiral yolk-shell nanorods [30] have been prepared. Using chiral biomolecules such as DNAs, amino acids, and proteins as the template, the achiral nanomaterials can be assembled into chiral nanostructures [31–38]. All these chiral materials were prepared without using circularly polarized light.

**Fig. 1:**
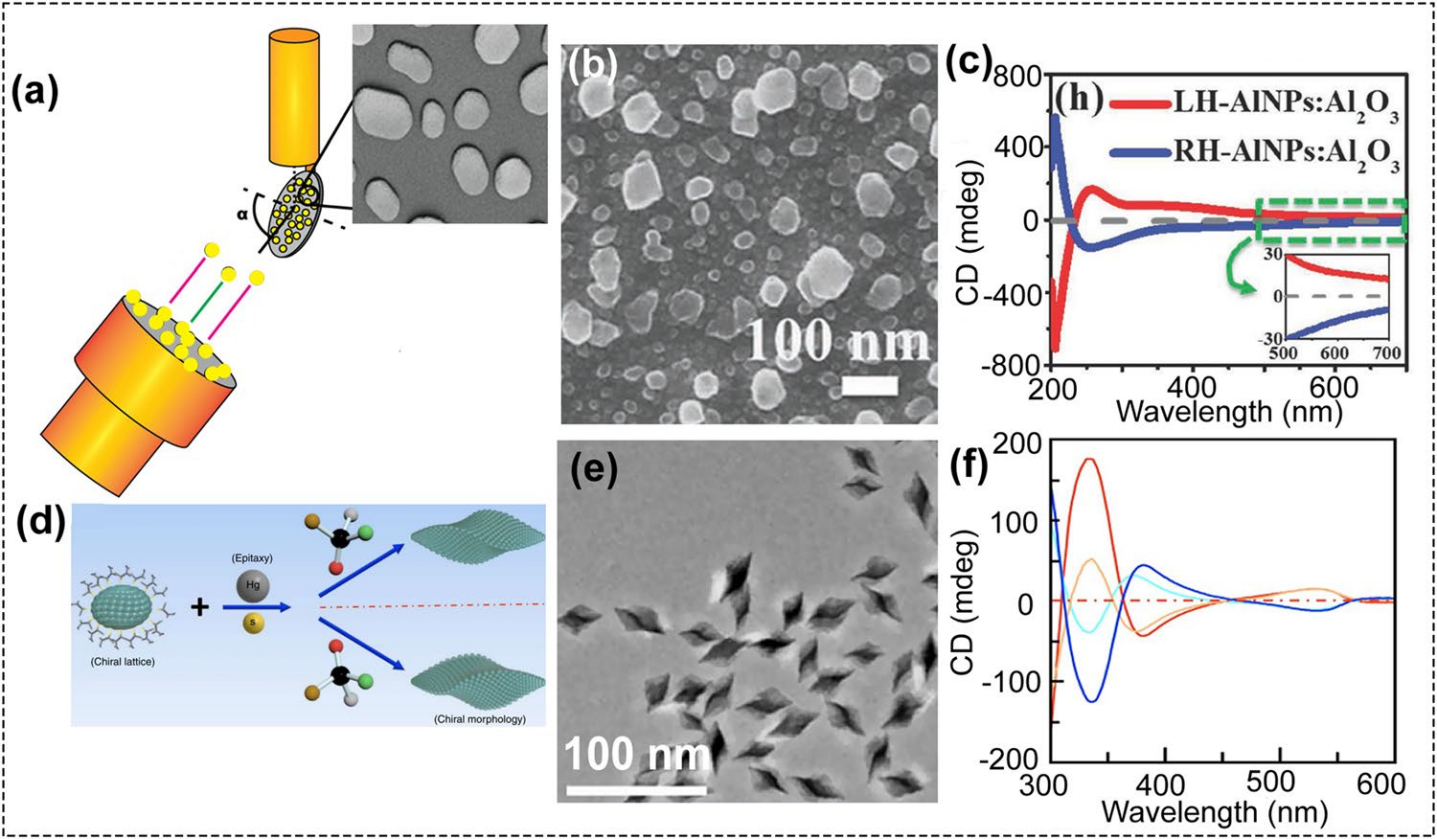
**a** Scheme of the glancing angle deposition (GLAD). **b** TEM image and **c** CD spectra of the chiral Al nanostructures prepared by GLAD (reproduced with permission from Ref. [40]. Copyright 2013, Wiley). **d** Scheme of the wet-chemical method to synthesize chiral HgS nanostructures. This method could allow the large-scale and cost-effective preparation of chiral nanostructures. **e** TEM image and **f** CD spectra of the chiral HgS nanostructures. Scale bar, 100 nm (reproduced with permission from Ref. [42]. Copyright 2017, Nature publishing group)

In addition, physical vapor deposition (PVD) and glancing angle deposition (GLAD) have been used to fabricate hybrid insulator–metal nanohooks [39] and chiral aluminum nanostructures [40], respectively (Fig. 1a–c). But these fabrication techniques are multi-step, complicated, and requires expensive, sophisticated equipment. Wet-chemical method, as a simple synthetic strategy, is becoming a promising method to create chiral nanostructures. For instance, inspired by the previous work [22, 41], the chiral HgS nanocrystals were prepared using D- or L-penicillamine molecules for surface reshaping in solution (Fig. 1d–f) [42]. For example, three-dimensional chiral plasmonic nanostructures with intense optical activity have been prepared by Luis’s group [10] and Nam’s group [11]. Lots of chiral materials, such as chiral nematic mesoporous materials [43], chiral liquid crystals [44], chiral helical structures [45, 46], chiral gold nanoclusters [47], chiral biointerface materials [48], and chiral plasmonic materials [48–51], have been prepared using wet-chemical method. Many comprehensive reviews on chiral materials have been published recently [50–63], most of them highlighted the synthetic strategy and optical properties of chiral materials, as well as their applications.

However, there are rare reviews that focus on the fabrication strategy using CPL as the chiral source to construct chiral materials. Given the fast development of this field, in this review, we summarize the recent advances of chiral materials (including chiral organic compounds and chiral inorganic structures) prepared with CPL and discuss their multifunctional applications.

## CPL-induced Asymmetric Synthesis

### Chiral Nanostructures Fabricated by CPL

**Fig. 2:**
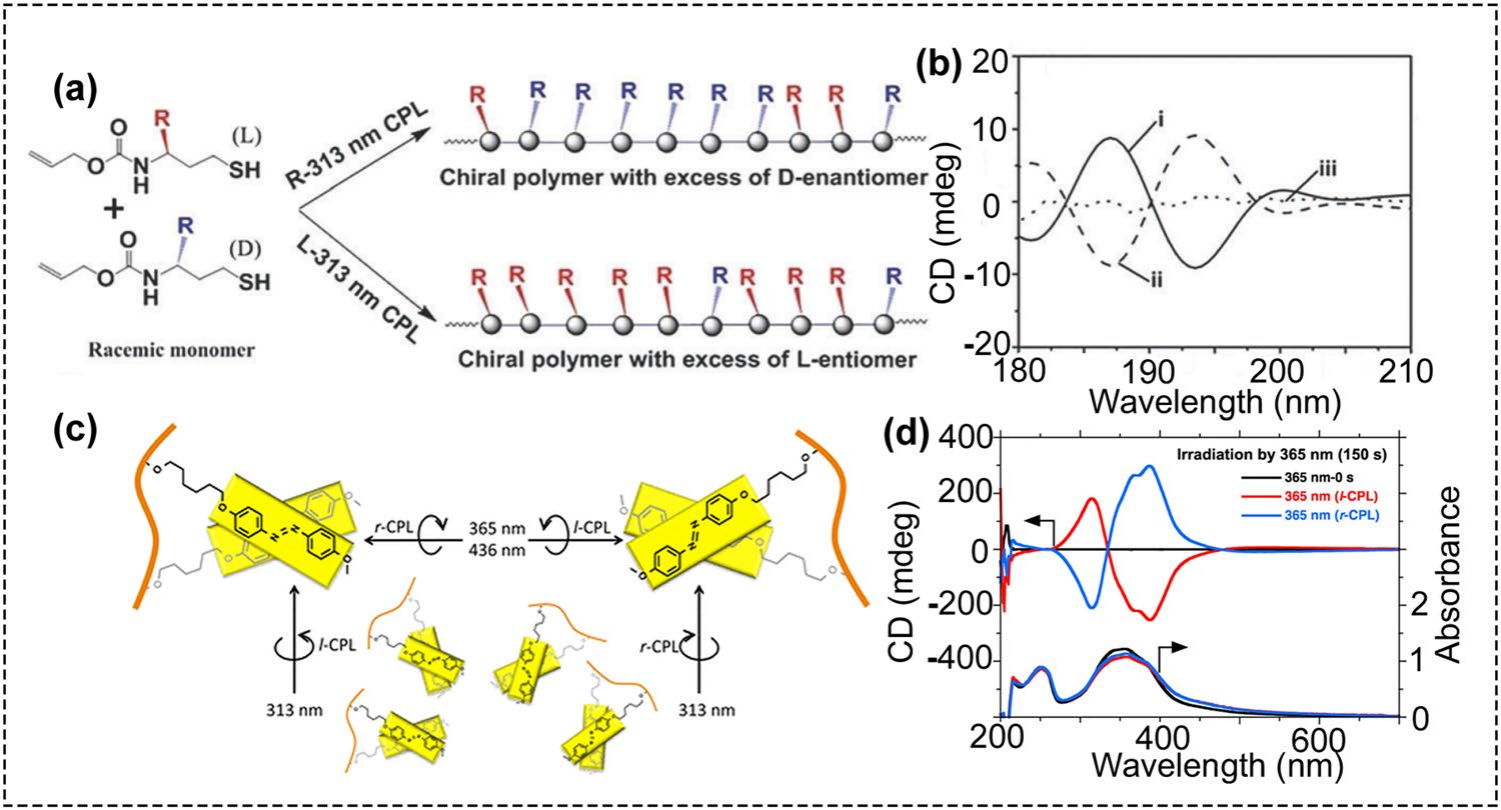
**a**, **b** Circularly polarized light triggered enantioselective thiol–ene polymerization reaction. **a** Schematic illustration of the growing optically active polymer from racemic monomers through the asymmetric thiol–ene polymerization process triggered by 313 nm CPL irradiation. **b** CD signals (irradiation for 90 min) of the specific rotation values of the final polymer obtained by irradiation with 313 nm (i) LCP, (ii) RCP, or (iii) normal UV light (reproduced with permission from Ref. [66]. Copyright 2017 the Royal Society of Chemistry). **c**, **d** CPL to regulate azobenzene supramolecular chirality. **c** Scheme of long- and short-axis-dependent twisted stack led by wavelength-dependent LCP and RCP light. **d** CD and UV–Vis spectra of PAzoMA 2 aggregates exposed to 365 nm LCP and RCP light for 150 s (reproduced with permission from Ref. [68]. Copyright 2017 the Royal Society of Chemistry)

Because light can be switched on and off rapidly, it offers high spatial and temporal resolution. The specific properties of CPL can provide a novel, powerful, versatile tool for enantioselective photochromism [64], enantiospecific desorption [65], chiral induction, symmetry breaking, and constructing chiral materials [65–74]. For example, Kim et al. applied CPL to a light-induced self-assembly process of a triphenylamine (TPA)-containing molecule. Mitsumasa IWAMOTO and cooperators reported the synthesis of chiral poly(diacetylene) (PDA) film polymerized from achiral monomers using CPL [74–77]. Under CPL irradiation, the enantioselective helical stacking of the TPA moieties promoted enrichment of one enantiomer, and produced the self-assembled aggregates with supramolecular chirality [78]. CPL was also utilized to trigger an enantioselective polymerization reaction, producing an optically active polymer from racemic monomers of allyl-(1-((3-(dimethylamino)propyl)amino)-4-mercapto-1-oxobutan-2-l)carbamate without using any chiral dopant or catalyst (Fig. 2a, b) [66]. CPL was also used to regulate the azobenzene supramolecular chirality. And the influence of CPL handedness, irradiation time, and wavelength on the resulting product’s chirality were systematically investigated (Fig. 2c, d) [68]. All these results provide new insight into the chirality of CPL-controlled molecules, supramolecules, and polymers.

**Scheme 1:**
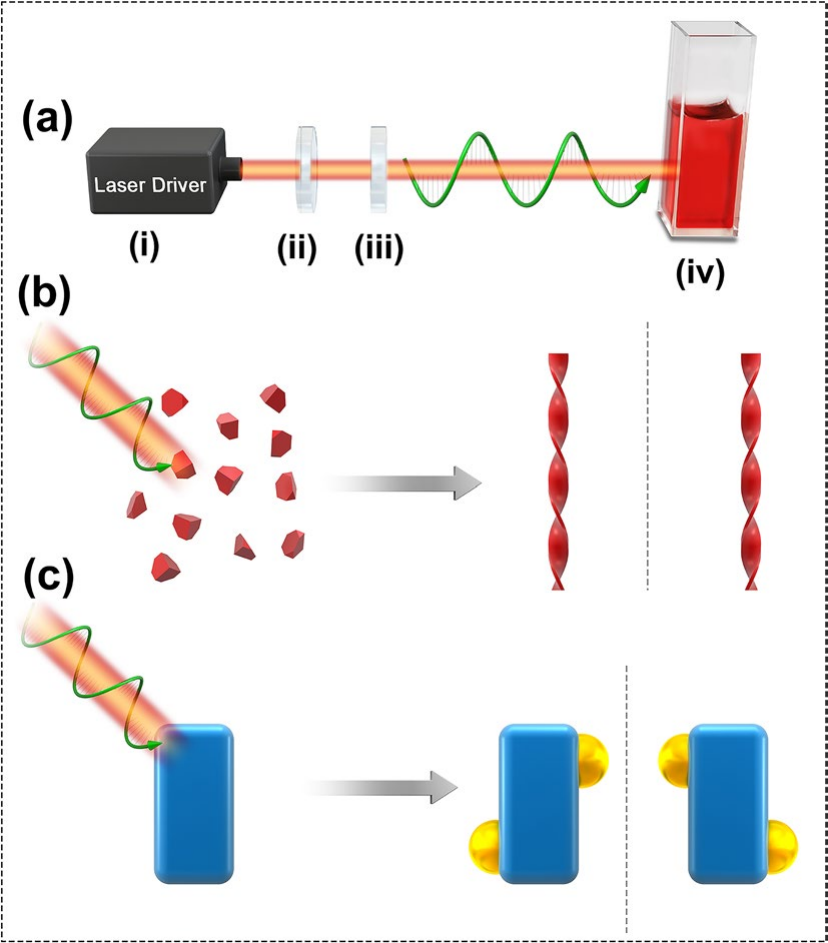
Experimental setup for CPL-driven synthesis. **a** CPL irradiation apparatus: (i) light source, (ii) polarizer, (iii) quarter wave plate, (iv) cuvette as reaction container. **b** CPL was used to drive the assembly of monodisperse nanoparticles into chiral helixes. **c** CPL was used to fabricate chiral inorganic nanostructures

Recent progress in using CPL to fabricate chiral nanostructures can be classified into two main strategies: CPL-driven self-assembly of nanoparticles into chiral structures and CPL-induced synthesis of chiral nanostructures (Scheme 1). Both strategies were based on the use of CPL as the chiral source in the growth reaction process. As shown in Scheme 1a, CPL is used to irradiate solutions of nanoparticle in a reaction vessel.

**Fig. 3:**
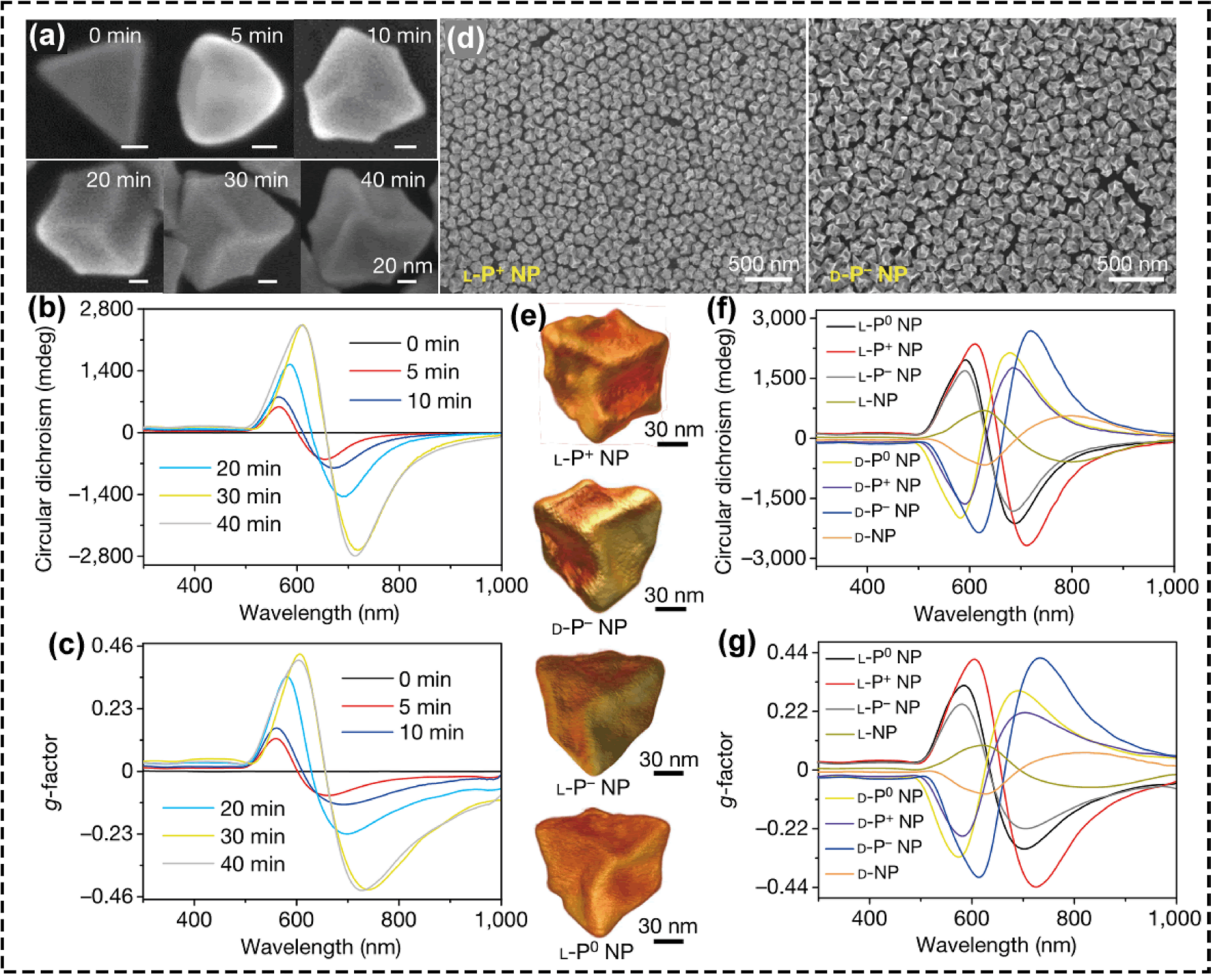
Morphology and spectroscopy of CPL-mediated chiral gold nanostructures. Scanning electron microscope (SEM) images **a**, circular dichroism spectra **b** and g-factor spectra **c** of L-P+ NPs after 0, 5, 10, 20, 30, and 40 min of illumination at 594 nm with 84 mW cm−2. **d** SEM images of L-P+ NPs and D-P− NPs. **e** TEM tomography images of L-P+, L-P−, D-P−, and L-P0 NPs. Circular dichroism spectra **f** and g-factor spectra **g** of NPs synthesized under different light conditions in the presence of CYP dipeptides: L-P+ NPs (under LCP illumination), D-P− NPs (under RCP illumination), D-P+ NPs (under LCP illumination), L-P− NPs (under RCP illumination), L-P0 NPs (under LP illumination), D-P.0 NPs (under LP illumination), L-NPs (without light illumination), and D-NPs (without light illumination) (reproduced with permission from Ref. [79]. Copyright 2022 Nature publishing group)

More recently, using trigonal nanoprisms as the seeds, our group synthesized chiral Au nanostructures with optical anisotropy factor (g-factor) of up to 0.44 under irradiation with CPL [79], which was the highest g-factor value up to now (Fig. 3). To discover the mechanisms underlying the CPL-mediated preparation of chiral Au nanostructures, the finite-difference time-domain (FDTD) and semi-empirical density functional theory (DFT) simulations of chiral Au nanostructure growth were carried out. The observed shapes of the chiral Au nanostructures can be explained by regioselective gold deposition on dynamically changing hotspots and localized reduction of Au(iii) to Au(0). As the electrical field is strongly localized in the corners of trigonal nanoprisms, the shape of the forming chiral Au nanostructure was depended by the handedness of CPL. Using iterative modeling for progressive deposition of gold on gradually changing hotspots, the final shape with out-of-plane Au segments was successfully modeled, which matched the key features of chiral Au nanostructure shape characterized by transmission electron microscopy (TEM) tomography. To further confirm the growth mechanism for the preparation of chiral Au nanostructure induced by CPL, other chiral gold nanomaterials such as gold nanocubes and octahedrons were also used as the seeds to prepare chiral nanostructures. The obtained chiral Au nanostructures also displayed remarkably high chiroptical activity under CPL illumination, indicating that the CPL-driven synthetic strategy is good to obtain chiral nanostructures with high g-factors.

Significantly, a very impressive result was obtained by Kotov and co-workers, who reported the synthesis of chiral twisted nanoribbons by illuminating the dispersion of racemic CdTe NPs with LCP or RCP light [80]. CdTe NPs were stabilized by the achiral capping agent thioglycolic acid (TGA). It should be noted that, this TGA capped CdTe dispersion showed no CD responses and thus has equal cumulative absorbance for LCP and RCP photons. Under LCP illumination, predominantly left-handed nanoribbons were formed, while right-handed nanoribbons were formed by RCP illumination. The obtained chiral nanoribbons were very stable and can retain their geometry for 3.5 years. Notably, illumination with unpolarized light generated the equal amount of left-handed and right-handed nanoribbons. Straight achiral nanowires were obtained by incubation in the dark or under linearly polarized light irradiation. Significantly, a control experimental demonstrated that D- and L-cysteine protected chiral CdTe NPs can self-assemble in the dark, generating submicron helices with distinctly different twist directions that was dependent on the chirality of D- and L-cysteine stabilized CdTe NPs.

The mechanism of chirality transfer from CPL to NP assemblies can be understood as follows. The effect of CPL on TGA capped CdTe NPs self-assembly originates in the optically selective activation of nanostructures with different handedness. The original CdTe NP solution is racemic, containing equal amounts of left- and right-handed particles and small clusters. After illuminating the solution with RCP light, a subpopulation of right-handed CdTe NPs and clusters absorb light more effectively than left-handed CdTe NPs and clusters. If illuminating the solution with LCP light, a subpopulation of left-handed CdTe NPs and clusters absorb light more effectively than right-handed CdTe NPs and clusters. The surface TGA ligands on the CdTe NPs were photo-oxidized and transformed into ‘bare’ CdS NPs. Because the photooxidation of multiple TGA ligands requires multiple photons, the difference in the probability of absorption of L- and R-photons multiplies over time. This process ‘locks in’ and amplifies the differences between NPs of opposite chirality in the initially racemic mixture. The bare CdS NPs were much easier to self-assemble than TGA-modified, non-light-activated CdTe NPs. The self-assembly of NPs is very sensitive to the anisotropy of NP interactions, thus the chirality of the constituent building blocks is reflected in the helicity of the resulting assemblies. Atomistic molecular dynamics (MD) simulations were performed to further clarify the origin of the helical nanoribbons. At first, the right-handed or left-handed CdTe NPs were preassembled into a planar piece of nanoribbon with a packing, assuming that NPs of predominantly one handedness were prepared under CPL photoexcitation and self-assembled. On equilibration of the NP assemblies in an isothermal–isobaric ensemble at T = 300 K for ∼ 5–10 ns, the planar nanoribbons acquired obvious twists. Notably, the twist was opposite for NPs with opposite handedness. The average twist angle observed in the simulation of the nanoribbons made from CdS NPs was − 3.1° and + 4.3° for left-handed and right-handed NPs, respectively, which corresponds to a pitch length of ∼1,400 – 1,900 nm, similar to the experimental pitch length of nanoribbons observed after 28 h CPL illumination. The MD simulations indicate that the chirality of the individual NPs translates into a twist of the nanoribbons as a result of cooperative interactions with the NP ensemble. Besides unequal truncations, this phenomenon may be associated with other chiral geometries and multiple interparticle interactions. Water molecules facilitate this process via the formation of a soft ‘cushion’ layer between NPs, enabling their restricted mobility. Translation and reorientation of NPs led to the possibility of ensemble-energy minimization in accordance with the chiral bias. The experimental structures are partially disordered because of fluctuations in NP size, which translate into some variability of the pitch and of the non-close packing of the NP lattice in the ribbon.

**Fig. 4:**
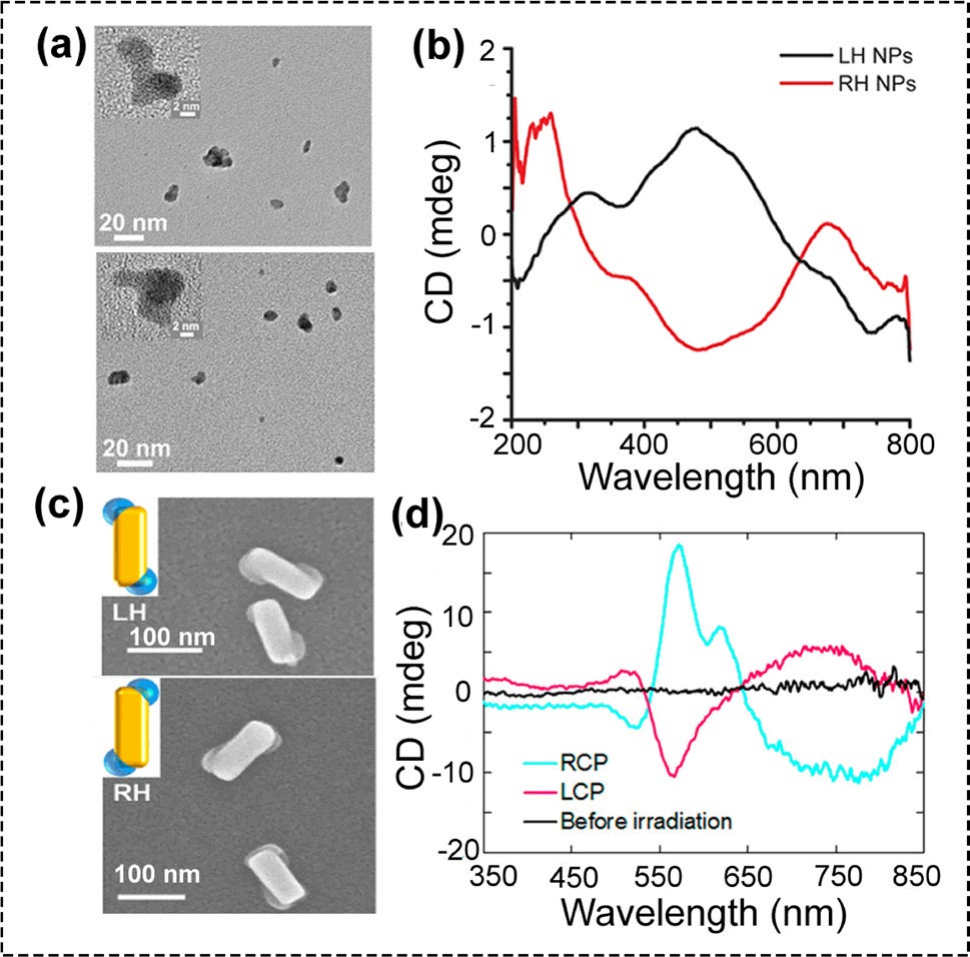
**a** TEM images and **b** of the chiral LH and RH AuNPs, which were prepared under the irradiation of LCP and RCP light, respectively (reproduced with permission from Ref. [81]. Copyright 2019 American Chemical Society). **c** SEM images of the chiral LH and RH Au-PbO2 nanostructures. **d** CD spectra of the TiO2 substrate with gold nanocuboids before (black line) and after PbO2 deposition by RCP (blue line) or LCP (red line) light irradiation (reproduced with permission from Ref. [82]. Copyright 2018 American Chemical Society)

The recent studies from Kotov’s group reported the assembly of gold nanoparticles into chiral superstructures by illumination of gold salt solutions with RCP or LCP light at 543 nm (Fig. 4a, b) [81]. After CPL irradiation for 50 min, the gold NPs were firstly formed and then assembled into chiral nanostructures, which showed a clear CD peak at 550 nm. Compared with twisted CdTe ribbons or helixes, these gold nanostructures did not show obvious chiral geometry, as shown in the tomography images. Through calculations of chiroptical properties, the percentage of chiral gold nanostructures among the prepared Au nanostructures was estimated to be 11.9% and 7.10% under LCP and RCP light illumination, respectively. This CPL-driven assembly of chiral gold nanostructures was originated from the asymmetric displacement of NPs in dynamic assemblies by plasmonic fields followed by particle-to-particle attachment. After CPL illumination, the chirality of photons was transferred to the chirality of Au NP assemblies. Transient interparticle forces, which were depended on the polarization of incident light, generate the chiral bias in the intermediate dynamic assemblies. Subsequently, the chiral bias was locked in shape via the integration of the nanoscale cores, resulting in the final chiral nanostructure. This synthetic protocol that using CPL-induced forces to prepare chiral plasmonic nanomaterials may be applicable to other dispersions, which were capable of spontaneous assembly into a superstructure with lattice-to-lattice connectivity.

Besides the synthesis of chiral materials in solution, the CPL-driven synthesis strategy can be extended to create chiral inorganic nanomaterials on the substrates. By employing circularly polarized light as the sole chiral source, Saito et al. prepared the chiral Au-PbO_2_ hybrid nanostructures with unique geometry on the TiO_2_ substrate (Fig. 4c, d) [82]. The optical activity of the chiral Au-PbO_2_ hybrid nanostructures was depended on the handedness of light. The enantiomeric excess value of the chiral Au-PbO_2_ nanostructures was as high as 43%. Under CPL irradiation, the Pb^2+^ in the solution was oxidized and then PbO_2_ was deposited at the specific corners of Au nanocuboids to form chiral Au-PbO_2_ hybrid nanostructures. Because the chiral structure was immobilized on a surface, thus the circularly polarized light was illuminated constantly from one orientation. Chiral deposition may be possible by the oxidation of plasmon-induced charge separation. When plasmonic nanoparticle (Au nanocuboids) was in contact with a semiconductor (TiO_2_), under CPL irradiation, the positive and negative charges are produced in the Au nanoparticle and TiO_2_, respectively, then Pb^2+^ in the solution was oxidized to PbO_2_ and deposited onto the corner of Au nanocuboids. These studies open up new ideas that the use of CPL as the primary chiral bias to prepare chiral inorganic nanostructures.

The various examples discussed in this part indicated that the CPL has the advantage of providing an easy and effective way to prepare chiral materials, allowing us to foresee that CPL approaches have a great potential toward the synthesis of chiral materials with tunable optical activity in solution or on the substrate. The mechanistic study showed that the CPL photoinduced site-selective reactions based on plasmon-induced charge separation resulted in the chiral nanostructures on the substrate. However, the mechanism of CPL-mediated preparation of chiral nanoparticles in solution is worthy to be further explored.

There are also some disadvantages about the CPL-induced synthetic strategy. Therefore, some chiral small molecules, helical polymers, supramolecular compounds, and liquid crystals after circularly polarized light illumination were observed. However, the e.e. for some of the reactions carried out using CPL is small, namely 0.1% − 2%. It is necessary to optimize the experimental condition and improve the e.e. value of chiral molecules. For the CPL-driven synthesis of chiral plasmonic superstructures, combining CPL-driven synthesis with the well-developed wet-chemical strategy, it could be used to synthesize more unique chiral structures with intense optical activity that cannot be prepared using conventional methods. More recently, combining CPL-driven synthetic strategy with the conventional strategy of chiral-ligand induced optical activity, it is possible to synthesize chiral Au nanoparticles with optical anisotropy factors of up to 0.44 [79].

### Tailoring Chirality of Self-Assembled Nanostructures by CPL

**Fig. 5:**
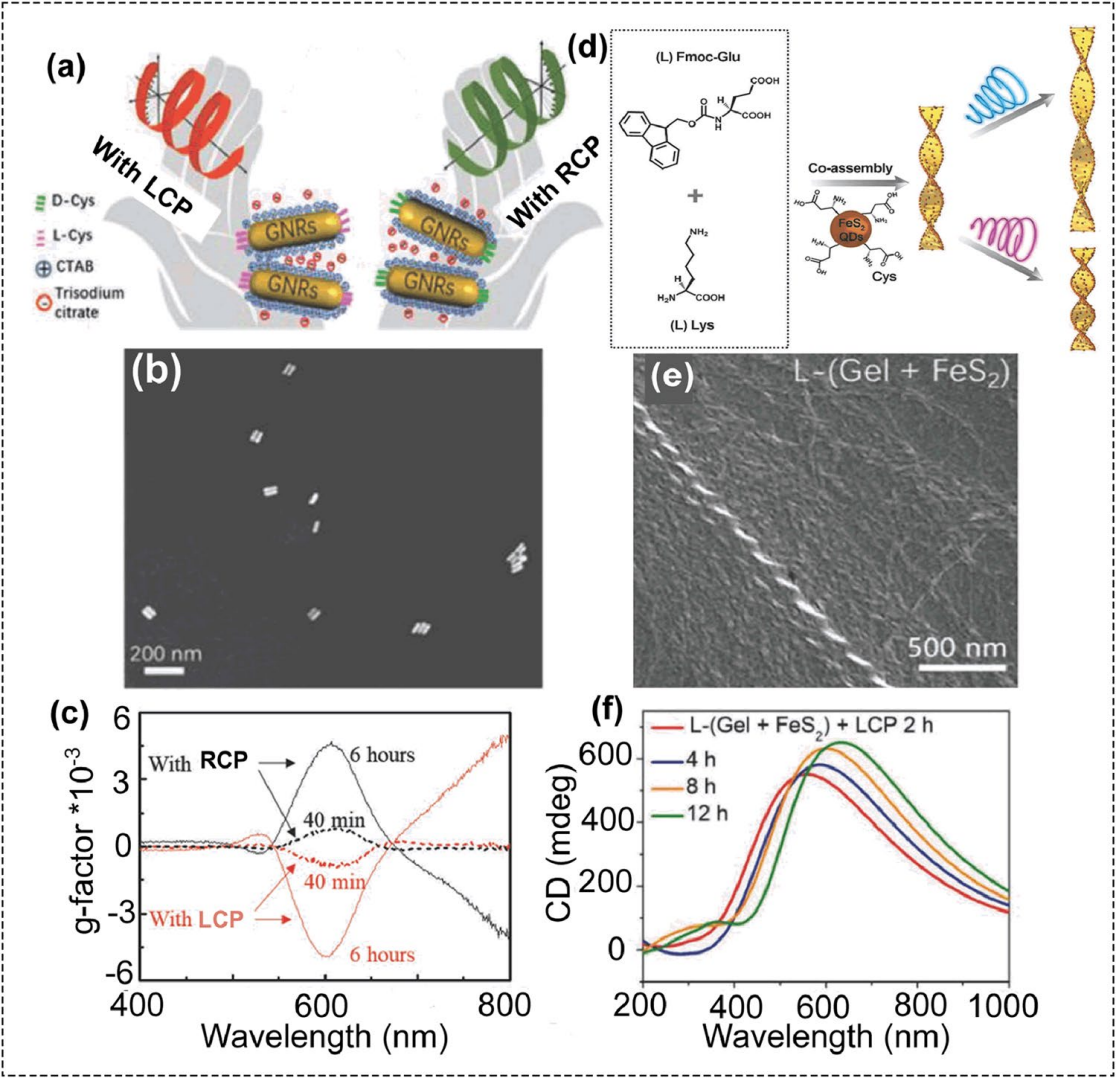
**a**–**c** Chiral GNR dimers and the asymmetric interactions with CPL. **a** Schematic illustrating the chiral GNR dimers with opposite handedness under illumination of CPL. **b** Typical SEM image of the GNR dimers. **c** g-factor spectra acquired from the GNR dimers under LCP or RCP light illuminations for 40 min (dashed lines) and 6 h (solid lines), respectively. LCP or RCP light was irradiated from a diode laser with a wavelength of 633 nm and a power density in the range of 50–90 mW cm^−2^. (reproduced with permission from Ref. [3]. Copyright 2019, WILEY–VCH GmbH). **d** Illustration of the co-gel formation and its CPL responsiveness. **e** SEM image of the L-(Gel + FeS_2_. **f** CD spectra of the L-(Gel + FeS_2_) under LCP light illumination for 2, 4, 8, and 12 h (reproduced with permission from Ref. [84]. Copyright 2019, WILEY–VCH GmbH)

Control of dynamic chirality in self-assembly systems is of great significance in exploitations of artificial nanomachines. DNA origami has been reported to achieve reconfigurable chiral assemblies with dynamic chirality. However, using DNA origami was relatively complicated. Recently, CPL was used to achieve optically chiral controls based on chiral gold nanorod (GNR) dimers (Fig. 5a-c) [3]. Two gold nanorods modified with chiral L- or D-cysteine (Cys) molecules were assembled into chiral L- or D-GNR dimers through electrostatic interactions. The chirality of GNR dimers was originated from the dihedral angle between the two GNRs. The L- and D-GNR dimers displayed a positive and negative CD peak centered at about 680 nm, which are referred to as P_L_- and M_D_-isomers, respectively. Illuminating P_L_-isomers with LCP light and M_D_-isomers with RCP light resulted in the inversion of CD response of P_L_-isomers and M_D_-isomers.

While almost no change was observed for illumination P_L_-isomers with RCP light or illumination M_D_-isomers with LCP light, indicating the CD change was dependent on the polarization states of CPL. Theoretical simulations showed that under CPL photoexcitation, the chiral GNR dimers can generate large optical torques, which caused this chiral switching. In addition to the optomechanical perturbation, the CPL-controlled chiral plasmonic nanosystems may also involve photochemical effect at the interfaces. This needs a further study to deeply understand the underlying mechanism and to explore other kinds of photoinduced chiral switching strategies. By increasing or decreasing the aspect ratio of GNRs, this CPL-driven photoswitches could work with different frequencies of light.

CPL can also be used to adjust the pitch of chiral helical structures. For helical nanostructures, their chiroptical properties were also determined by their helical pitch length. Thus, tuning the pitch of chiral helical structures is the key to adjust the optical properties. Using a series of peptide conjugate molecules, Rosi’s group prepared a family of chiral helical gold nanoparticle single helices, their pitch can be systematically adjusted by the length of peptide aliphatic tails [83]. Significantly, the pitch and diameter of the hydrogel can also be tuned by LCP or RCP light (Fig. 5d–f) [84]. The hydrogel was consisted of chiral iron disulfide quantum dots (FeS_2_ QDs) and two gelators (N-(9-fluorenylmethoxycarbonyl)-protected L/D-glutamic acid, Fmoc-L/D-Glu and chiral L/D-lysine, L/D-Lys) [84]. The chiral FeS_2_ QDs with an average diameter of 5 nm were prepared using L/D-cysteine (Cys) as the capping ligands. Then, the chiral L/D-FeS_2_ QDs in water were mixed with Fmoc-L/D-Glu and L/D-Lys, a helical co-gel L/D-(Gel + FeS_2_) was formed after heating the mixture and cooling down to the room temperature. The co-gel L/D-(Gel + FeS_2_) exhibited intense optical activity and circularly polarized luminescence. The responsiveness of the L/D-(Gel + FeS_2_) to CPL light (532 nm, 78 mW cm^−2^) was investigated. After illuminating D-(Gel + FeS_2_) with LCP light, the helical pitch was increased, while the pitch was decreased after illumination with RCP light. For L-(Gel + FeS_2_), the pitch was increased after illumination with RCP light, whereas the LCP light will cause the decrease in pitch value. Besides helical pitch, the helix diameter of the L/D-(Gel + FeS_2_) was also changed after illumination with CPL. For D-(Gel + FeS_2_), the helix diameter was increased after illumination with RCP light, while the LCP light will cause the decrease in helix diameter. All the data indicated that the degree of twisting (twist pitch) and the diameter of the co-gels can be regulated by illumination with CPL.

## CPL-Activated Applications Based on Chiral Materials

Chiral materials have great application prospect in biosensing, asymmetric catalysis, and biomedicine. Given the fact that CPL can be used to prepare chiral materials, we envision that chiral materials could have more unique and useful applications under the help of CPL. In this section, we will discuss the CPL-activated promising applications based on chiral materials, with a primary focus in the fields of biosensing, combating bacteria, circularly polarized photocatalysis, and phototherapy. Although some of these applications can also be implemented in the absence of CPL, we must emphasize that the CPL could further improve the effect on the basis of its exceptional chiroptical performance.

### CPL-Triggered Biosensing

**Fig. 6:**
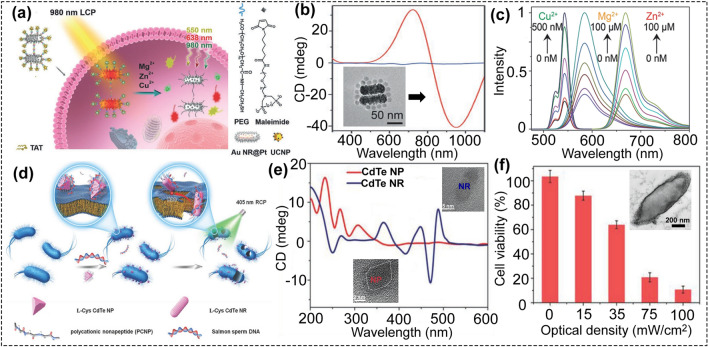
**a** Schematic illustration of the Au NR@Pt dimer-UCNP satellites for intracellular triple-ion detection. **b** CD spectra of the Au NR@Pt dimer-UCNP satellites, and the inset is the typical TEM image of the Au NR@Pt dimer-UCNP satellites. **c** Normalized fluorescence intensity of satellite assemblies with various concentrations of the Cu2+, Zn2+, and Mg.2+ ions (reproduced with permission from Ref. [86]. Copyright 2019, WILEY–VCH GmbH). **d** Schematic illustration of the chiral CdTe NPs for combating gram-negative bacteria under CPL illumination. **e** CD spectra of the CdTe NPs and the CdTe NRs, and the inset is the typical TEM image of CdTe NP and NR. **f** Cell viability of E. coli incubated with chiral CdTe NPs and then treated with different illumination intensities (405 nm, 30 min). The inset is the TEM image of the E. coli after treating with chiral CdTe NPs and CPL (reproduced with permission from Ref. [89]. Copyright 2018, WILEY–VCH GmbH)

Based on chiral assemblies, CPL can also be applied in the field of biosensing. By employing metal-dependent DNAzymes [85], a chiral core-satellite nanoprobe was developed to detect multiple metal ions in live cells under CPL irradiation (Fig. 6a-c) [86]. The chiral core-satellites nanostructures consist of a spiny platinum coated with gold nanorod dimer (Au NR@Pt, core) and several up-conversion nanoparticles (UCNPs, satellites). Through DNA hybridization, Au NR@Pt and UCNPs were assembled into the chiral Au NRs@Pt-UCNPs probe. Significantly, the intracellular CD response of Au NRs@Pt-UCNPs core-satellite assemblies was reversed after being uptake by the HeLa cells. This interesting property could enable the Au NRs@Pt-UCNPs to be used as intra/extracellular biosensors under irradiation of CPL. The key role of CPL was to increase the local temperature of the chiral assembly. After illumination with 980 nm LCP light (5 Mw cm^−2^, 2 min), the temperature of chiral core-satellite assemblies could reach 57 °C, which is higher than the melting temperature of the metal-ion-protected substrate DNA, resulting in the release of the protected DNA strands from the assemblies. Notably, the other light conditions such as RCP light cannot cause the similar temperature change. In the presence of target metals, the fluorescence signals of Cy5, TAMRA, and UCNPs, which were quenched by Au NRs@Pt, could be restored. And this chiral nanoprobe showed good linear relationships when applied for sensing Zn^2+^, Mg^2+^, and Cu^2+^. This new method of CPL-activated biosensor could be extended to probe other significant targets in live cells.

### Combating Bacteria

Multidrug-resistant bacteria are becoming increasingly present in humans, animals, and the environment, and pose a serious threat to public health that needs to be addressed as soon as possible. Chiral materials are widely investigated in biosensing owing to their unique optical properties. Recently, Sun et al. reported that chiral heterodimers of UCNP and gold yolk–shell NP can be applied for quantitative analysis and imaging of antibiotic-resistant bacteria in vivo [87]. Interestingly, under CPL illumination, chiral cadmium telluride (CdTe) NPs can also be good candidates to combat bacteria. The concept is that chiral CdTe NPs will transform into nanorods under CPL illumination (405 nm, 100 mW cm^−2^), which was consistent with the previous report [88]. The nanorods can destroy of the bacteria membrane, about 93% *E. coli* were dead after 30 min. At the same time, a large amount of reactive oxygen species (mainly hydroxyl radical) was generated during the transformation of NPs to NRs, which could cause the serious damage of the membrane and then kill the bacterial (Fig. 6d-f) [89]. Moreover, due to the strong fluorescent property, the chiral CdTe NP was the excellent fluorescent probe and can be used for the fluorescence imaging-guided bacterial infection therapy. This study opens up a new way for using both CPL and chiral nanomaterials to treat bacterial infection.

### CPL-Triggered Catalysis

**Fig. 7:**
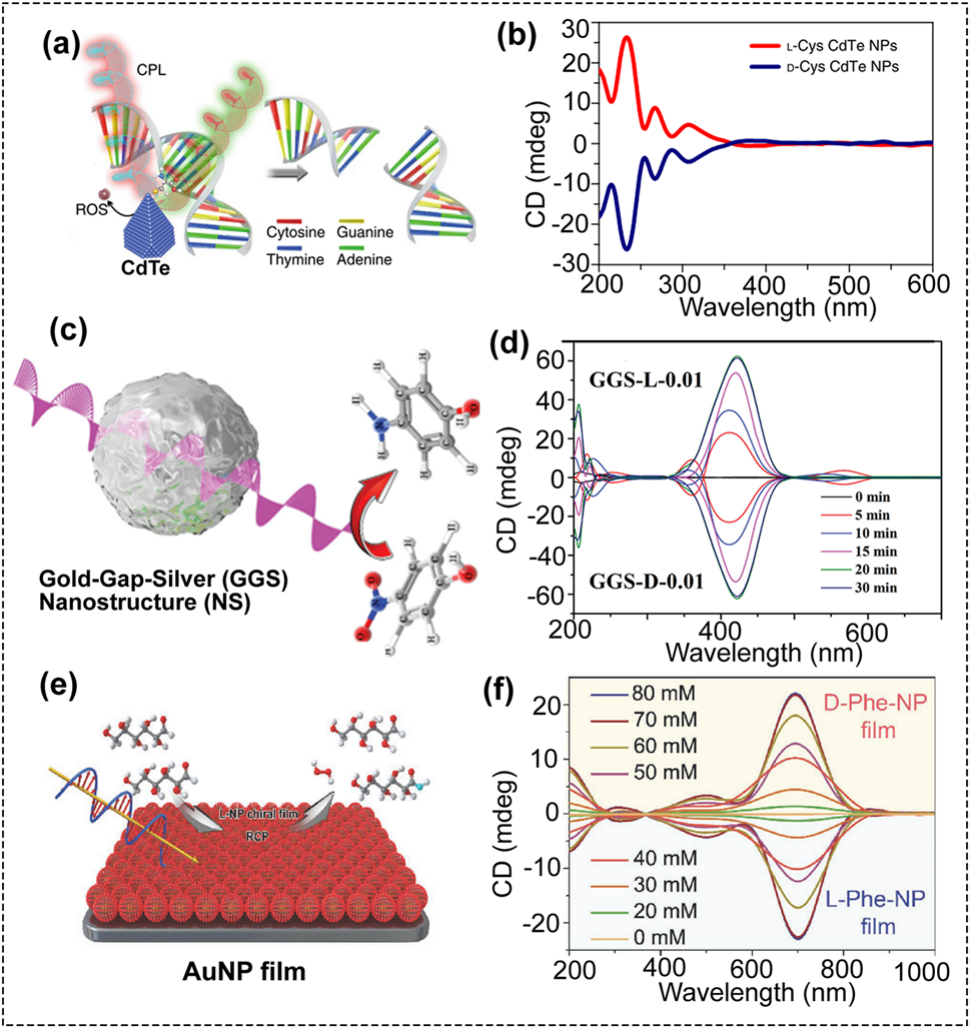
**a** Schematic illustration of chiral CdTe-based specific DNA cleavage under CPL irradiation. **b** CD spectra of the chiral CdTe NPs (reproduced with permission from Ref. [88]. Copyright 2018 Nature publishing group). **c** Schematic illumination of the photocatalytic reduction of 4-nitrophenol by chiral Gold-Gap-Silver (GGS) nanostructures (NS). **d** CD spectra of GGS nanostructures prepared at different reaction times (reproduced with permission from Ref. [90]. Copyright 2015, WILEY–VCH GmbH). **e** Schematic illumination of photocatalytic oxidation of glucose enantiomers by chiral AuNP film. **f** CD spectra of the chiral AuNP films (reproduced with permission from Ref. [92]. Copyright 2018, WILEY–VCH GmbH)

As a specific light, CPL can be used to accelerate and even trigger some photocatalytic reactions. For instance, Sun et al. demonstrated that chiral CdTe quantum dots (QDs) with a unique truncated tetrahedral shape could be used as artificial restriction endonuclease (Fig. 7a, b) [88]. This chiral CdTe QDs with tetrahedral shape were prepared using D/L-cysteine as chiral ligands, and the diameter was 4.5 ± 0.3 nm. CD spectra showed that these chiral QDs showed mirror signals between 350 and 410 nm. The prepared chiral QDs can be used to specifically recognize and cut double-stranded DNA (dsDNA) at the site GAT′ ATC (′ shows the cut site) under CPL illumination. It was found that the strong affinity between chiral Cys and the conformation of the specific DNA sequence induced the specific sequence selectivity. More importantly, these CdTe QDs can be used for cutting DNAs in living cells and in nude mice. The mechanism study showed that photoinduced reactive oxygen species (ROS) are responsible for DNA cleavage. Simultaneously with DNA cleavage, the ROS content in the DNA solution increased in response to light over time. Moreover, under the same exposure time, the ROS production by the chiral L- or D-CdTe QDs was clearly affected by the different polarization directions. L-QDs produced more ROS under RCP light than under LCP light. Consistent with the ROS yield, L-QDs showed a higher cleavage rate under RCP than under LCP light irradiation. This difference can be attributed to the differential absorption efficiency of the chiral CdTe QDs under CPL. Therefore, chiral CdTe QDs showed specific nuclease-mimetic activity under the corresponding CPL. Although photoinduced ROS are responsible for the cleavage activity, the sequence selectivity arises from the affinity between chiral cysteine ligands and the conformation of the specific DNA sequence, as confirmed by quantum-chemical calculations. This is the first report that using chiral inorganic nanoparticles as the artificial endonuclease.

In a recent report, chiral copper sulfide QDs were prepared in the presence of chiral L-/D-Pen and used to cut proteins under the illumination of circularly polarized light. Notably, the L-type QDs showed the highest catalytic performance under the illumination of left-circularly polarized light. Similar to the above chiral CdTe QDs, mechanistic studies demonstrated the production of hydroxyl radicals under CPL illumination that resulted in the cutting of proteins [90].

In addition, CPL can also be used to accelerate the catalytic reaction using chiral plasmonic gold-gap-silver (GGS) nanostructures as the catalyst (Fig. 7c, d) [91]. The chiroplasmonic GGS nanostructures with interior gaps were enantioselectively fabricated using L/D-cysteine as the chiral ligands, denoted as GGS-L/D. The plasmonic CD response of GGS-L/D could be adjusted by the concentration of chiral cysteine in the synthetic process. Significantly, under illumination with LCP light, the catalytic efficiency of converting 4-nitrophenol to 4-aminophenol by GGS-L is about fivefold, tenfold, 11-fold, and 12-fold higher than those of the photocatalytic reactions irradiated by linearly polarized (LP), RCP, natural light, and without light, respectively. The reason why LCP light resulted in the highest catalytic efficiency was that LCP light activated a larger number of GGS-L than other light conditions, which increased the number of hot electrons of chiral plasmonic gold-gap-silver nanostructures by adsorbing the related rotation of polarized light. Moreover, the CPL could activate hot electrons of chiral plasmonic nanostructures, resulting in the much higher catalytic activity. This work opens up avenues for using chiral plasmonic nanostructures to chiral catalysis.

Besides the colloidal chiral materials, chiral plasmonic film could also be used for catalysis under CPL irradiation (Fig. 7e, f) [92]. Chiral Au NP films that modified with chiral D-/L-phenylalanine (Phe) molecules (denoted as D/L-Phe-NP films) can be used as the photo-oxidant under CPL irradiation for selective oxidation of glucose enantiomers. For D-glucose oxidation, when using L-Phe-NP films as the catalysts, the kinetic rate constant was 2.14 min^−1^ under RCP light illumination, which was about 2.9-, 7.6-, and 21.4-fold higher than those of the photocatalytic reactions irradiated by linearly polarized (LP) light, LCP light, and dark condition, respectively. All the results showed that the catalytic performance was light-polarization dependent. Significantly, the optical activity of the chiral film was almost unchanged after five times cyclic reaction, suggesting that this chiral NP film has good stability and could be reused.

The reasons for the polarized light-enhanced photocatalysis with high selectivity are as follows. Firstly, L-Phe and D-Phe onto the surface of Au NPs can self-assemble to chiral arrangement, which was very important in the chiral selection of glucose enantiomers. The chiral arrangement will make L-Phe-modified Au surface has higher catalytic activity toward the oxidation of D-glucose molecules, and will make the D-Phe-modified Au surface has higher activity toward the oxidation of L-glucose molecules. Secondly, chiral AuNP films selectively absorb photons of specific light handedness, resulting in matter–photon interaction. For instance, L-Phe-NP films showed a much stronger propensity to absorb LCP light at 660 nm. Excited plasmons on the gold surface act to populate O_2_ antibonding orbitals and to form a transient negative-ion state to boost the oxidation reaction of glucose. Thirdly, CPL containing all polarization angles within one optical period can activate numerous NPs, thus increasing the light absorption efficiency. Fourthly, due to the efficient absorption of CPL, the rate of plasmon formation in the hotspot region is markedly accelerated with maximized local electromagnetic field enhancement in the hotspot region. This is owing to surface plasmonic resonance, which greatly increases the catalytic rates. Fifthly, the intensity of local photons in the hotspot region could be enhanced elastic scattering of the incident light by adjacent NPs and by increasing the photon path lengths and steady-state photon intensity. The energy of the elastic scattering photon could accelerate the oxidation.

### CPL-Triggered Cell Manipulation

**Fig. 8:**
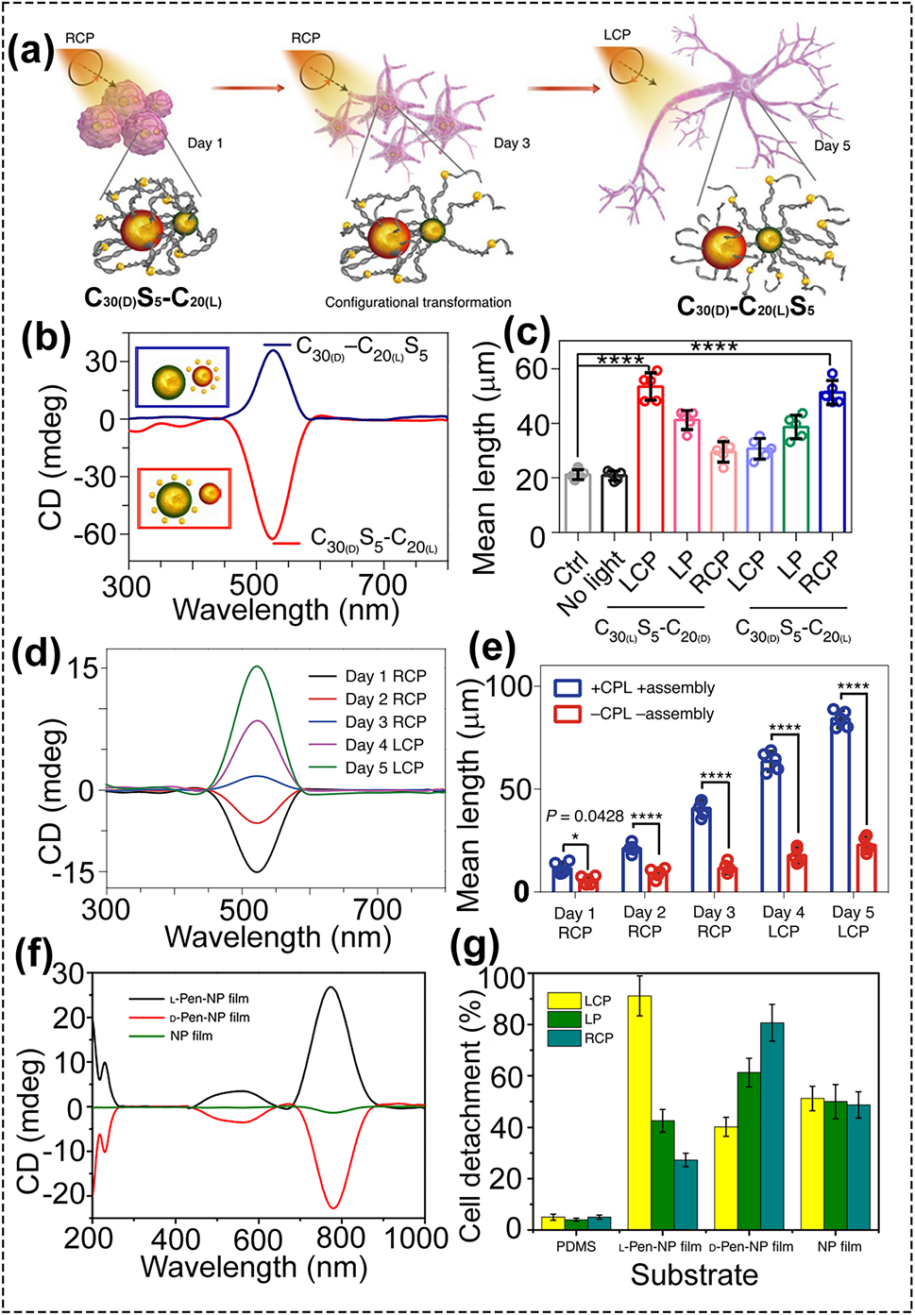
**a** Schematic of differentiation of NSCs with CPL after daily incubation with C30(D)S5-C20(L) for five days. **b** CD spectra of C30(D)S5-C20(L) and C30(D)-C20(L)S5 in phosphate buffered saline. **c** Neurite mean length of differentiated NSCs incubated with C30(D)S5-C20(L) or C30(D)-C20(L)S5 for 4 h each day, and subsequently illuminated with CPL (50 μJ per pulse, 50 Hz, 5 min) for five days, or incubated with C30(D)S5-C20(L) without illumination for 5 days; cells without nanoassemblies or light exposure were used as a control. **d** Mean lengths of neurites in differentiated NSCs. **e** CD spectra of differentiated NSCs from day 1 to day 5 (reproduced with permission from Ref. [93]. Copyright 2021 Nature publishing group). **f** CD spectra of Au NP film and L/D-Pen modified Au NP film. **g** Cell detachment rates upon different light irradiation (reproduced with permission from Ref. [94]. Copyright 2017 Nature publishing group)

The biological effects of CPL on living cells are considered to be negligibly weak. However, under the help of the chiral assemblies and circularly polarized photons, differentiation of neural stem cells into neurons can be accelerated (Fig. 8a–d) [93]. Au NPs of three different sizes (5, 20, and 30 nm) were coated with chiral D-/L-cysteine (D-/L-Cys) and then assembled into chiral NP assemblies by DNA hybridization, denoted as C_30(D)_S_5_-C_20(L)_. In the presence of Fox3, C_30(D)_S_5_-C_20(L)_ will turned into C_30(D)_-C_20(L)_S_5_. The C_30(D)_S_5_-C_20(L)_ showed negative CD peak, while C_30(D)_-C_20(L)_S_5_ displayed positive CD response. After incubating C_30(D)_S_5_-C_20(L)_ with neural stem cells (NSCs), the effects of photonic polarization on the NSCs differentiation with RCP, LCP and linearly polarized (LP) light (532 nm, 50-Hz pulse rate, 5 min each day for five days) were studied. RCP light illumination caused the largest increase in neurite length compared with other light conditions (LCP, LP). The mechanism of CPL-accelerated NSC differentiation was also studied. The difference in NSC differentiation between these illumination conditions was due to the different light-dependent force on the cytoskeleton under different light illuminations. The plasmonic-force calculations showed that the C_30(D)_S_5_-C_20(L)_ assembly exerted nine times larger force under RCP illumination than the force under LCP and LP illumination. To test the functionality of CPL-differentiated NSCs, the obtained neurons were implanted in the hippocampus of a mouse model of Alzheimer’s disease. After treatment, the amount of amyloid-β (Aβ) and hyperphosphorylated tau (p-tau) proteins in AD mice were reduced by more than 70%. This work indicated that the biological effects of CPL can be applied to cellular development for biomedical use.

Using light to manipulate cells has obvious advantages and has captured the attention of investigators. For cell detachment, using near-infrared (NIR) light can avoid the irreversible damage caused by the digestive enzymes. But NIR light can lead to the increase in temperature, which was bad for the cells. To avoid the heating effect and improve the efficiency of light in cell manipulation, CPL was introduced. Chiral Au NP film with intense optical activity can be applied to promote cell adhesion, growth, differentiation, and retrieve cells without damage under CPL (Fig. 8e, f) [94]. The chiral Au NP film was consisted of achiral Au NPs and chiral L/D-penicillamine (denoted as L/D-Pen-NP film). The NG108-15 cells were chosen as the model cells. On the surface of chiral L-Pen-NP film, the rate of cell proliferation was accelerated, while the rate was slowed on the D-Pen-NP film. Interestingly, CPL (808 nm laser) can be used to significantly improve the cell detachment efficacy without cell damage. Under the optimized condition (LCP light, 150 mW cm^−2^ for 5 min), the detachment percentage of cells on the L-Pen-NP film was 91.2 ± 7.8%. No heating effect was observed after illumination, and the viability of cells retrieved from L-Pen-NP film exceeded 95%, which was much higher than the cells retrieved from the D-Pen-NP film. This could be owing to that more proteins were adsorbed on the L-Pen-NP film, which might work as a protecting layer, and this strategy for cell detachment by remote irradiation with CPL was even safer than the digestive enzymes.

### CPL-Triggered Phototherapy

Photodynamic therapy (PDT) is a noninvasive, site-selective way with minimal side effects that has emerged as an efficient treatment for local cancer cell ablation. Although a huge amount of photosensitizers have been used as PDT agents for cancer therapy, their limited ROS-generating capability caused low PDT efficiency. This issue can be addressed using chiral materials and CPL. For example, chiral dimers of AuNPs modified with protoporphyrin IX (PpIX) could enable dichroic targeting of PDT under CPL illumination [95]. The chiral dimers were assembled via DNA hybridization and displayed scissor-like geometry with a distinctive dihedral angle. Significantly, the intracellular AuNP dimer showed a mirror structure of the extracellular dimer during transmembrane transport. Correspondingly, the plasmonic CD responses changed from negative to positive after entering cells. For PDT experiment, the chiral AuNP dimers were incubated with HeLa cells, then the cells were illuminated with different light conditions. The viability of cells was dramatically decreased when circular polarization of incident photons matched to the preferential absorption of chiral dimers localized inside the cells, which is associated with the increased generation of reactive oxygen species (ROS). LCP light resulted in a three-time higher cell mortality rate compared with RCP or LP light. Significantly, the difference between LCP and RCP light illumination in apoptosis induction for chiral AuNP dimers was much greater than one might expect based on the intracellular concentration of photosensitizers, g-factor, and the difference of light absorption by the twisted AuNP dimers. This is quite surprising from the optical standpoint and a detailed investigation needs to be performed to fully understand the origin of such strong PDT effect. The ROS generation experiments showed that the amount and localization of ROS are the key in understanding the biological effect of RCP and LCP photons. The large difference between RCP and LCP photons is specific to the concentration of the photodynamic therapy agents and the chiral AuNP dimers.

To further prove the elimination of cancer cells, the chiral gold nanorod (GNR) dimers modified with Chlorine6 (Ce6) were used to confirm the effect of circular polarization in killing cells. The polystyrene-block-poly(acrylic acid) (PS-PAA) was used to coat on the GNR dimers, and then, the intracellular CD signal was the same to the extracellular CD response. Due to the chiral GNR dimers showed a negative CD peak, so the RCP light gives a highest efficiency in killing cancer cells. Therefore, the chiral dimers of AuNP and GNR proved the dichroic targeting of cancer cells incorporating of chiral nanostructures with specific handedness.

**Fig. 9:**
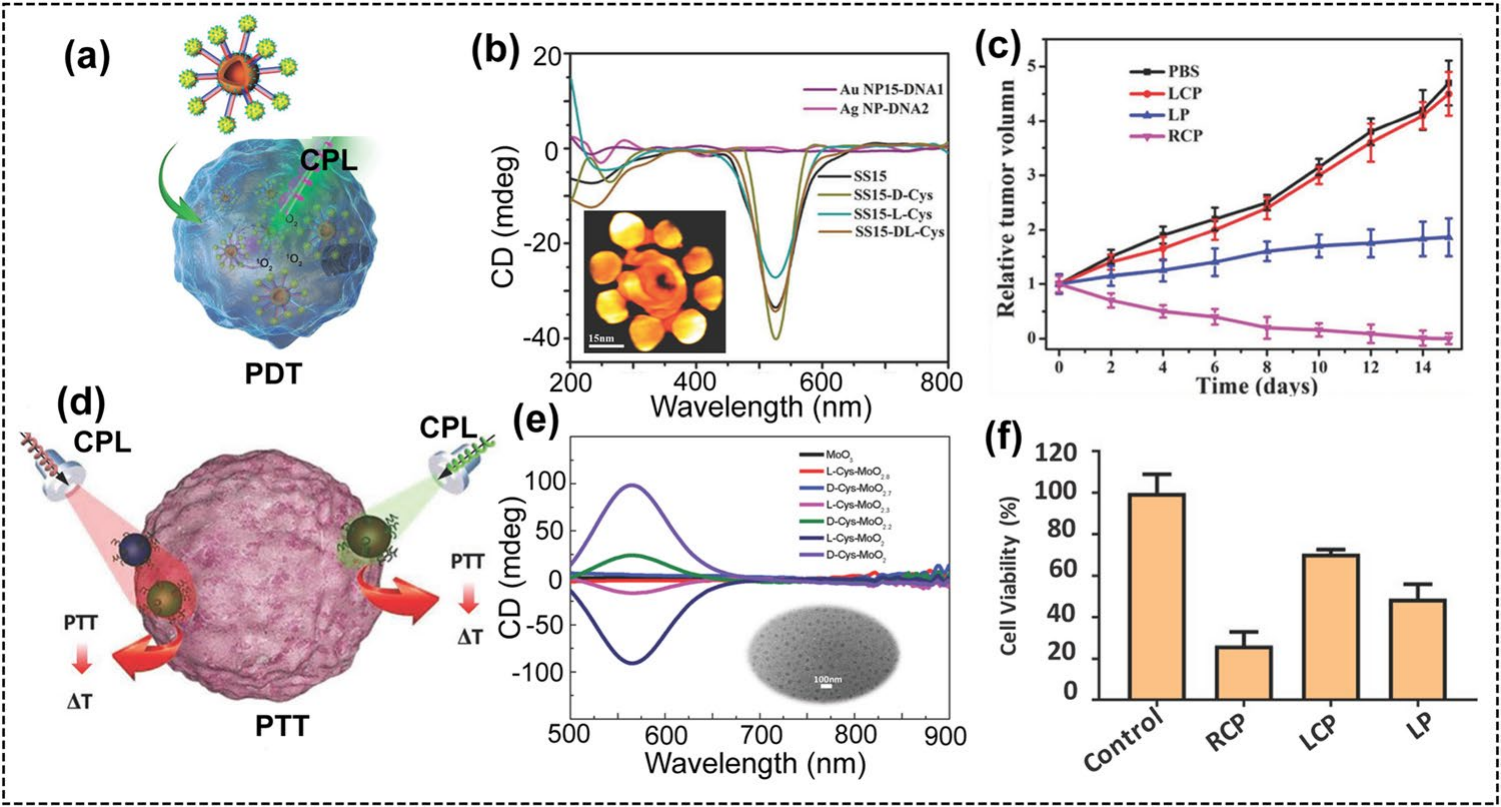
**a** Illustration of self-assembled shell–satellite (SS) nanostructure as a chiral photodynamic therapy agent under CPL illustration. **b** CD spectra of the chiral SS15 nanostructure. The inset is the 3D tomography of the SS15 nanoassembly. **c** The relative tumor growth curves after various treatments: PBS only, SS15 assembly + LCP light, SS15 assembly + LP light, and SS15 assembly + RCP light (reproduced with permission from Ref. [96]. Copyright 2017, WILEY–VCH GmbH). **d** Scheme for the synthesis of Cys-MoO3−x NPs and their applications for tumor cell ablation via CPL radiation. **e** CD spectra of chiral Cys-MoO3−x NPs. **f** Viability of HeLa cell incubating with chiral D-Cys-MoO2 (50 µg mL−1) after 532 nm RCP, LP, and LCP irradiation (1 W cm.−2 for 15 min) was analyzed by CCK-8 assay (reproduced with permission from Ref. [97]. Copyright 2019, WILEY–VCH GmbH)

To further enhance the efficiency of using chiral structures for PDT, chiral shell–satellite (SS) assemblies were developed as PDT agents to treat cancer (Fig. 9a–c) [96]. The Ag NP (core) and Au NPs (satellites) were assembled to form the core–satellites assemblies. Next, chloroauric acid was added to transfer the core–satellites into shell–satellite (SS) assemblies. Then chiral D-/L-cysteine was modified onto the SS assemblies, forming the chiral plasmonic SS-D/L-Cys assemblies. These chiral SS-D/L-Cys assemblies, as chiral photosensitizers, can generate ROS under CPL irradiation. Under LCP light irradiation, SS-L-Cys assemblies produced the higher degree of ROS than SS-D-Cys assemblies due to the higher energy transfer efficiency. Under illumination with RCP light, SS-D-Cys generated more ROS than the SS-L-Cys, and even higher than protoporphyrin IX (PpIX), the classic organic photosensitizer. In order to study the phototherapeutic potential, chiral SS assemblies were incubated with HeLa cells for 24 h and then removed the excess assemblies. Under RCP irradiation, SS-D-Cys assemblies killed more cancer cells than SS-L-Cys assemblies. As a control, the achiral PpIX showed much weaker phototherapeutic effect and no light preference was observed. The therapeutic efficacy of the SS-D-Cys assemblies was evaluated in vivo using a nude mouse tumor model. Under RCP light irradiation, the tumor was completely eliminated and no regrowth of tumor was observed, indicating that the PDT was highly efficient for tumor treatment under RCP.

In a recent report, under CPL irradiation, chiral nanomaterials were also used as photothermal therapy (PTT) agents to eradicate cancer cells. Using chiral cysteine as the ligands, chiral substoichiometric molybdenum oxide (L/D-MoO_3−*x*_) nanoparticles with intense CD response in the visible‐ and near‐infrared‐light regions were prepared (Fig. 9d-f) [97]. This chiral L/D-MoO_3−*x*_ could be applied as the photothermal therapy agent for tumor cell ablation. Under illumination with LCP light, L-MoO_3−*x*_ showed the highest efficiency for killing HeLa cells, while the D-MoO_3−*x*_ gave the highest mortality for HeLa cell ablation under RCP light, indicating the chiral selectivity of L/D-MoO_3−*x*_ for incident light. Such chirality-dependent photothermal therapy of chiral MoO_3−*x*_ NPs can be ascribed to the differential absorption efficiency of the chiral NPs under different CPL illumination. When CPL was used as light source, chiral MoO_3−*x*_ NPs can generate stronger local heating effects than LP light irradiation, which will cause higher cancerous cell killing rate.

## Conclusion and Outlook

In this review, we firstly summarized the recent progress on the preparation of chiral materials using circularly polarized light (CPL). As a chiral source, CPL has been found in star-forming regions, and could be a possible origin of single chirality in nature. By locking the chirality of the incident photons, CPL was used to induce enantiomeric excess from a prochiral photosensitive molecule (such as azobenzene dimer [98]), produce chiral porous solids containing only achiral building blocks [99], and synthesize chiral coordination polymer [100]. Moreover, CPL could transfer the chiral information from photons to the materials, which has been used for the preparation of chiral organic compounds (such as chiral polymers and amino acid derivatives) and inorganic nanostructures, and CPL can regulate the dynamic chirality at molecular level and in chiral nanoassemblies. On the other hand, circularly polarized light can be applied to many fields based on the chiral materials. By using chiral assemblies, a CPL-activation method was proposed for detecting the metal ions in live cells. Also CPL can be applied for combating bacterial, phototherapy, catalysis, cell manipulation, and so on.

On account of the above-mentioned progress of CPL-enabled chiral nanomaterials, there is plenty of room for explorations to further advance this exciting multidisciplinary field. Bottleneck problems still remain and need to address in the future. For instance, how can the optical responses of CPL-induced chiral structures be enhanced? How can CPL be used to further improve the yield of chiral nanomaterials with high yield for stronger optical responses? When both CPL and chiral ligand are used in the synthesis of chiral materials, does chiral ligand play any essential role in the synthetic processes? These are very relevant open questions that certainly deserve a lot of brilliant ideas and experimental efforts.

The development of CPL-driven synthetic approaches to obtain chiral nanoparticles with high morphological and chemical stability, as well as strong optical activity, can expand their applicability in different technologies. For the CPL-triggered catalysis, such as chirality-dependent photocatalytic water splitting using CPL, the detailed mechanism under these reactions was still unclear. This requires more comprehensive mechanistic study to fully elaborate the phenomena. In the future, optimized material combinations and morphology control with better matched CPL excitation have potential to achieve much higher performance of chirality-sensitive photochemistry.

Because many kinds of inorganic nanoparticles (such as quantum dots and plasmonic nanomaterials) are light sensitive, using CPL to fabricate chiral structures should have a broad prospect. For now, most studies are still focused on Au, but other plasmonic metals, such as silver, aluminum, or palladium, will be worth exploring. With the deeper understanding of interactions of CPL and chiral materials and the emerging of advanced characterization methods, we envision that the CPL-activated chiral materials could provide more important and unprecedented applications in detection, biomedicine, biocatalysis, and life science.
